# Lettuce Chlorosis Virus Disease: A New Threat to Cannabis Production

**DOI:** 10.3390/v11090802

**Published:** 2019-08-29

**Authors:** Lior Hadad, Neta Luria, Elisheva Smith, Noa Sela, Oded Lachman, Aviv Dombrovsky

**Affiliations:** 1 Department of Plant Pathology and Weed Research, Agricultural Research Organization, The Volcani Center, 68 HaMaccabim Road, P.O.B 15159, Rishon LeZion 7505101, Israel; 2 The Robert H. Smith Faculty of Agriculture, Food and Environment, The Hebrew University of Jerusalem, Rehovot 761001, Israel

**Keywords:** *Closteroviridae*, *Crinivirus*, *Bemisia tabaci*, chlorosis, shoot propagation

## Abstract

In a survey conducted in *Cannabis sativa* L. (cannabis) authorized farms in Israel, plants showed disease symptoms characteristic of nutrition deprivation. Interveinal chlorosis, brittleness, and occasional necrosis were observed in older leaves. Next generation sequencing analysis of RNA extracted from symptomatic leaves revealed the presence of lettuce chlorosis virus (LCV), a crinivirus that belongs to the *Closteroviridae* family. The complete viral genome sequence was obtained using RT-PCR and Rapid Amplification of cDNA Ends (RACE) PCR followed by Sanger sequencing. The two LCV RNA genome segments shared 85–99% nucleotide sequence identity with LCV isolates from GenBank database. The whitefly *Bemisia tabaci* Middle Eastern Asia Minor1 (MEAM1) biotype transmitted the disease from symptomatic cannabis plants to un-infected ‘healthy’ cannabis, *Lactuca sativa,* and *Catharanthus roseus* plants. Shoots from symptomatic cannabis plants, used for plant propagation, constituted a primary inoculum of the disease. To the best of our knowledge, this is the first report of cannabis plant disease caused by LCV.

## 1. Introduction

*Cannabis sativa* L. (cannabis) had been used throughout the history of humankind for industrial purposes and traditional medicine worldwide [1]. In recent years, the medicinal properties of cannabis plants have been globally re-acknowledged. This was supported by studies on the cannabinoids synthesized by the plants [2] and their impact on various medical conditions [3]. The new findings have led to legislation change and permits were issued allowing cultivation of cannabis plants for medical purposes in many countries [4,5,6,7]. However, plant pathology research and studies of the appropriate management of cannabis cultivars are still in progress. Nowadays, in the authorized farms (AF) in Israel, medicinal cannabis is cultivated as a monoculture crop, which is highly susceptible to disease spread [8]. Furthermore, cannabis plants are propagated via ‘mother plants’ shoots, which could preserve and initiate spread of pathogens [9]. Several old studies on viral diseases in cannabis cultivars were reported including the diseases caused by hemp streak virus (HSV) [10] and hemp mosaic virus (HMV) [11]. However, these studies lack disease cause diagnoses, which are based on serology and molecular biology [12]. Recent reports on cannabis diseases have shown the occurrence of cucumber mosaic virus (CMV), alfalfa mosaic virus (AMV), arabis mosaic virus (ArMV) [12] and the viroid hop latent viroid (HLVd) [13,14] in cannabis plants. In addition, a cryptic virus, cannabis cryptic virus (CCV) had been isolated from cannabis [15]. In the AF in Israel, cannabis is cultivated primarily for medical purposes and therefore the use of chemicals for pest control is limited. The consequence of the limited use of pesticides is the increase in plant infestation by mites and various known insect vector species e.g., aphids, thrips and whiteflies. In recent years, whitefly-transmitted viruses have become a major risk for vegetable crop production, reducing quantity and quality of various crops [16,17]. In Israel, whitefly transmitted virus species belonging to the *Begomovirus*, *Crinivirus* and *Ipomovirus* genera have been recently identified in mixed infections of watermelon fruit samples [18]. The objective of the current study was to assess the presence of viral diseases in medical cannabis grown in AF in Israel in order to establish the pest management protocols for the cannabis industry.

## 2. Materials and Methods

### 2.1. Collection of Symptomatic Cannabis Plants

**Figure 1:**
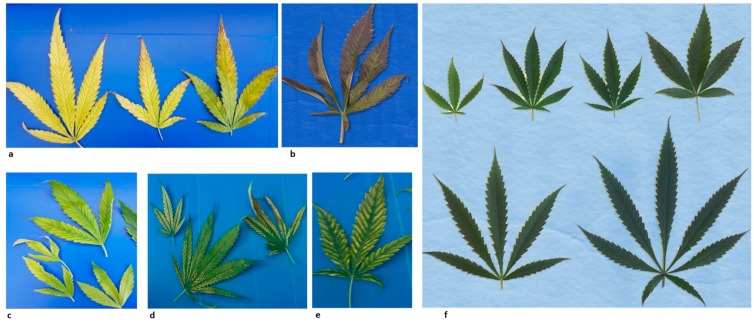
Disease symptoms of lettuce chlorosis virus on old leaves of cannabis plants at the vegetative stage. (**a**) Yellowing leaves showing necrosis. (**b**) Purple leaves. (**c**) Chlorotic leaves. (**d**) Interveinal yellowing leaves showing necrosis, (**e**) Interveinal yellowing leaves. (**f**) Cannabis leaves of uninfected ‘healthy’ leaves.

A survey was conducted in commercial cannabis AF located at the North, center, and South of Israel, during the years 2017–2019, in order to identify symptoms of suspected viral diseases. In four out of seven farms, conspicuous symptoms of yellowing, chlorotic, and necrotic foliage were observed (Figure 1). The symptomatic plants were collected for further investigation.

### 2.2. Small RNA Extraction, Next-Generation Sequencing (NGS)-Illumina MiSeq and Bioinformatics Analysis

Small RNA (sRNA) extraction and NGS followed by bioinformatics analysis were conducted as previously described [18]. Small RNA was extracted from symptomatic cannabis leaves pooled from six plants, using mirVana miRNA Isolation Kit (Invitrogen/Thermo Fisher Scientific, Waltham, MA, USA). The enriched small RNA was subjected to miRNA library construction using TruSeq Small RNA Sample Preparation Kit (Illumina, San Diego, CA, USA) as described previously [19]. Illumina MiSeq sequencing platform was used for library sequencing at the Agriculture Research Organization (ARO), The Volcani Center. For data analysis, sRNA WORKBENCH [20] was used for removal of the 3′ adaptor from the raw reads. Low quality reads were filtered using Trimmomatic v0.32 [21]. The VirusDetect software, (Cornell, NY, USA) version 1.7 was used for identification of viral contigs from the clean sequences [22]. Then, bowtie2 software (Johns Hopkins, Baltimore, MD, USA) was used for depth calculation and whole virus genome coverage [23] and the SAMtools (MIT, Boston, MA, USA) was used for mapping [21].

### 2.3. Total RNA Extraction, NGS-Illumina HiSeq and Bioinformatics Analysis

Total RNA extraction and NGS followed by bioinformatics analysis were performed as previously described [18]. In general, total RNA extracted from symptomatic cannabis leaves of one plant, subjected to ribosomal RNA depletion, served for library construction using ScriptSeq™ Complete Kit (Plant Leaf, Illumina, San Diego, CA, USA). The library was sequenced using Illumina Hiseq 2500 (fifty cycles), (Technion Genome Center, Haifa, Israel). Obtained clean reads were searched for matched viral sequences using VirusDetect software version 1.7 with the plant virus database and default parameters of the software pipeline [22]. The assembly used by VirusDetect software involved a pipeline combining de-novo assembly with mapping to references of plant viruses from Genbank using velvet [24] and Burrows-Wheeler Alignment tool (BWA) [25] for mapping reads.

### 2.4. Genome Assembly of the Cannabis Lettuce Chlorosis Virus (LCV) Isolate

Symptomatic cannabis leaves from 10 plants served for enrichment of viral RNA using AccuPrep Viral RNA (Bioneer, Daejeon, South Korea). The obtained viral RNA served as a template for cDNA synthesis by Reverse transcription (RT) reaction using Maxima Reverse Transcriptase cDNA kit (Thermo Fisher Scientific, Waltham, MA, USA). Reverse complement primers used in the RT reaction, were designed according to the obtained NGS results of LCV: RNA1-R-8433 (5′-GTTACCAGCCTTGAGTCAATCA-3′) and RNA2-R-8189 (5′-TGAACAATCACTACAGGTTTGG-3′). The obtained cDNAs were amplified in a polymerase chain reaction (PCR) using JMR PCR master mix (JMR Holdings, Kent, UK) with specific primer pairs designed based on the NGS results (Table S1, primer sets 1–13). The obtained amplicons were sequenced by Sanger sequencing (HyLabs, Rehovot, Israel) and sequences were aligned with California LCV RNA1 and RNA2 reference genome (accession Nos. FJ380118 and FJ380119). Additional primer pairs were designed based on the sequenced amplicons for complete sequencing of the cannabis isolate (Table S1, primer sets 14–22). The 5′ and 3′ untranslated region (UTR) sequence of the two segments was obtained using SMARTer RACE Kit (Clontech, Takara, CA, USA) and according to the manufacturer’s protocol we have deleted the SMARTer II A oligonucleotide as well as the five X nucleotides located at the 5′ [26] (Table S1, primers-RACE5′ and RACE3′). RACE obtained sequences served for primer design for the 5′ (RNA1-F-1 and RNA2-F-1) and 3′ prime ends (RNA1 and RNA2-R-3′), defining the first and last nucleotides in each segment of the genome (Table S1, primer sets 1, 6, 7 and 12). Sequence analysis was performed using the NCBI BLAST algorithms and Multalin version 5.4.1 was used for multiple sequence alignment. In addition, by referring to the attained NGS results, we have designed overlapping primer sets, six for each RNA segment, that cover the virus genome (Table S2).

### 2.5. Phylogenetic Tree Analysis

Phylogenetic tree analysis was performed based on the putative capsid protein (CP) amino acid sequence of six LCV isolates and several other criniviruses. The sequences were aligned by multiple sequence alignment using Muscle [27]. The closterovirus citrus tristeza virus (CTV) served as an out-group. The tree was constructed using MEGA6 software based on maximum likelihood method [28] with the parameter of 100 bootstrap.

### 2.6. Whitefly Transmission Experiments of the Cannabis LCV Isolate

Cannabis plants showing typical yellowing disease symptoms that were LCV positive in RT-PCR analysis were selected for the experiments. LCV -infected plant-branches immersed in water were placed in a plastic plant growing box [25× 30 × 30 cm box (with 50 mesh net attached to two box walls and the box cover)] and served as a source for the transmission experiments. The silverleaf whitefly *Bemisia tabaci*, Middle Eastern Asia Minor1 (MEAM1) biotype, propagated on *Gossypium hirsutum* (cotton) plants, were employed for two transmission experiments. Prior to the transmission experiments, samples of fifty and one hundred whiteflies were analyzed by RT-PCR to confirm that the whitefly population is LCV free. The whiteflies were placed inside a growth box containing symptomatic LCV-infected cannabis plant-branches for 24 h acquisition access feeding (AAF) period. The viruliferous whiteflies were then transferred to a new box containing un-infected cannabis plants (healthy, as confirmed by RT-PCR), kindly provided by Dr. Moshe Flaishman, ARO The Volcani Center, for 24 h allowing the inoculation access feeding (IAF) period. Following the IAF period, the plants were treated with insecticide mixture containing Verimark^®^ (DUPONT) and Confidor^®^ ( Bayer, Leverkusen, Germany). Following another 24 h, the insecticide-treated plants were transferred to a growth chamber, keeping a photoperiod of 18 h light, 6 h dark, and a temperature of 22 °C for symptom development at the vegetative stage. The presence of LCV was determined by RT-PCR using specific primers (Table S1, primer sets 1, 6, 7 and 13).

### 2.7. Partial Host Range of Cannabis LCV Isolate

Two LCV host plants were tested for their susceptibility to the cannabis LCV isolate. LCV-free (tested by RT-PCR) *Lactuca sativa* L. (lettuce) (cultivars Butter-head and Romaine) and *Catharanthus roseus* var. roseus (rose periwinkle) served for a partial host range experiment. Inoculation of the plants with the cannabis LCV isolate was performed by a whitefly transmission experiment described above. LCV inoculated plants were monitored for symptom development in an insect-proof growth chamber inside a greenhouse. Following leaf yellowing symptom appearance (at thirty days post-inoculation), leaf samples were collected for LCV analysis by RT-PCR (Table S1, primer sets 1, 5, 7 and 12). The experiment was repeated twice.

### 2.8. LCV Transmission via Cannabis Shoots

Study of LCV transmission through cannabis plant shoots was carried out by analyzing shoots from two cannabis plants of ca. four week old, one was LCV infected and the other was virus free (as confirmed by RT-PCR). Five shoots were separated from each ‘mother plant’ using a sterile scalpel, dipped in a hormone powder and placed in moist soil pots. The cannabis shoots grew for an additional eight weeks in the growth room under conditions similar to those described above, and monitored for symptom development. The plants were sampled and diagnosed for LCV infection by RT-PCR (Table S1 primer sets 5 and 12).

### 2.9. Study of Seed Transmission of the Cannabis LCV

One hundred seeds collected from symptomatic cannabis plants were sown for analysis of seed transmission ratio. Three hundred seeds collected from three symptomatic plants (one hundred seeds from each plant) were analyzed by RT-PCR for the presence of the cannabis LCV. The presence of the cannabis LCV in the pooled seeds and in eight seedlings (pooled) was analyzed by RT-PCR (Table S1 primer sets 8 and 13).

## 3. Results

### 3.1. A Survey

A survey was conducted in medical cannabis commercial authorized farms in Israel, during the years 2017–2019. Conspicuous foliage symptoms of yellowing, chlorotic and necrotic old leaves were observed with no apparent symptoms on the apical leaves (Figure 1). In the infected farms, thirty different genotypes were symptomatic. In 2017, three farms had the disease symptoms in 15–30% of the plants while the ‘mother plants’, which were used for propagation, were grown separately and were asymptomatic. About two years later, on May 2019, a fourth farm, showed disease incidence in 100% of the plants and the ‘mother plants’, grown separately, were infected as well. In order to uncover the causing agent of the cannabis yellowing disease, we applied the unbiased NGS analysis to reveal potential viral pathogen/s.

### 3.2. Next Generation Sequencing Analysis of Small RNA Preparation

**Figure 2:**
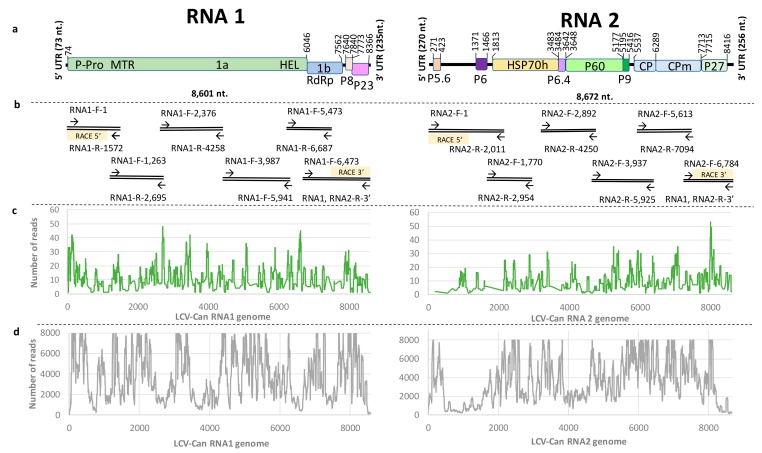
Genome organization of cannabis lettuce chlorosis virus (LCV) isolate. (**a**) A scheme of the cannabis LCV isolate RNA1 and RNA2 segments encoding putative viral proteins represented in rectangles. (**b**) RT-PCR primers that established the full genome sequence of the cannabis LCV isolate (Table S2). (**c**) Obtained reads and coverage of cannabis LCV isolate by NGS Illumina Miseq sequencing of small RNA preparation. (**d**) Obtained reads and coverage of cannabis LCV isolate by NGS Illumina Hiseq sequencing of total RNA preparation.

Samples of symptomatic cannabis leaves were collected from commercial cannabis AF for laboratory analysis (each sample for NGS originated from 10 sub-samples of symptomatic plants) (Figure 1). Small RNA, extracted from leaf samples, served for sRNA library preparation followed by NGS llumina Miseq sequencing. The raw data obtained contained 790,490 reads. After 3′ adaptor removal, 682,961 reads remained, which were used for length range filtering (16 to 35 bp), resulting in 446,593 reads. The reads were assembled into sixty short contigs containing 41–124 nt, that were mapped based on the reference genome sequence of California LCV (accession Nos._FJ380118 and FJ380119) (Figure 2). LCV belonging to the *Crinivirus* genus (*Closteroviridae* family) has a bipartite ssRNA genome (Figure 2a). Of the sixty viral contigs obtained by the NGS Illumina Miseq, twenty two contigs mapped to the RNA1 segment and thirty eight contigs mapped to the RNA2 segment, yielding coverage along the reference genome of only 12.3% and 24.4%, respectively (Figure 2c, Table 1).

### 3.3. Next Generation Sequencing Analysis of Total RNA Preparations

In order to improve the data obtained by NGS of sRNA, total RNA extracted from symptomatic cannabis leaves served for library construction and sequencing by Illumina Hiseq platform. The raw data contained 30,021,710 reads and No. of reads after filtering out low quality sequences was 1,541,368. The NGS data allowed assembly of 82.7% and 78.6% of RNA1 and RNA2 of the cannabis LCV isolate, respectively (Figure 2d, Table 1).

The bioinformatics analysis did not reveal the presence of any other virus or viroid in both the sRNA and total RNA preparations. Since cannabis-leaf-chlorosis has been recently attributed to the occurrence of the viroid hop latent viroid (HLVd) [14], we confirmed the bioinformatics results by RT-PCR of viral RNA prepared from both original samples that were previously analyzed by NGS, using the specific following primers: HLVdF and HLVdR [14] and the samples were negative for the viroid.

### 3.4. Revealing the Complete Genome Sequence of Cannabis LCV Isolate by RT-PCR Amplifications and Sanger Sequencing

Based on the obtained NGS data, RT-PCR amplifications were applied to attain and validate the complete genome sequence of the cannabis LCV isolate. The RACE technique was applied using SMARTer RACE kit (Clontech Laboratories, Takara, CA, USA) for retrieving the 5′ and 3′ ends after removal of SMARTer II A oligonucleotide including the five X nucleotides at the 5′. The obtained large segment amplicons (six for each RNA segment including the RACE amplicons) of ca. 2000 bp each (Figure 2) were sequenced using Sanger sequencing method. The obtained sequences were analyzed by BLAST algorithms and the final version of the genome was assembled by integrating the results of the NGS and the Sanger sequencing of the RT-PCR and the 5′ and 3′ RACE PCR (Figure 2). The complete genome sequence of the cannabis LCV isolate, provisionally named LCV-Can, was submitted to GenBank (accession Nos. MK747245 and MK747246).

### 3.5. Genome Organization of LCV-Can

LCV-Can genome, comprised of two fragments: RNA1 of 8601 nt, encoding four putative proteins (1a, RdRp, p8, p23) and RNA2 of 8672 nt, encoding nine putative proteins (P5.6, P6-like, HSP70h, P6.4, P60, P9, CP, CPm, P27) as depicted in Figure 2. BLASTn comparison results showed that LCV-Can shared high sequence identity with other LCV isolates (Table 2). Importantly, LCV-Can RNA1 and RNA2 shared 99.6% nucleotide sequence identity with *Phaseolus vulgaris* (bean) LCV isolate from Almeria Spain (accession Nos. MG489894 and MG489895), covering 90% and 99% of the genome, respectively. When compared to RNA1 and RNA2 of *L. sativa* LCV isolate from California, USA (accession Nos. FJ380118 and FJ380119) LCV-Can genome shared 88.5% and 90.4% nucleotide sequence identity covering 99% and 92% of the genome, respectively.

**Figure 3:**
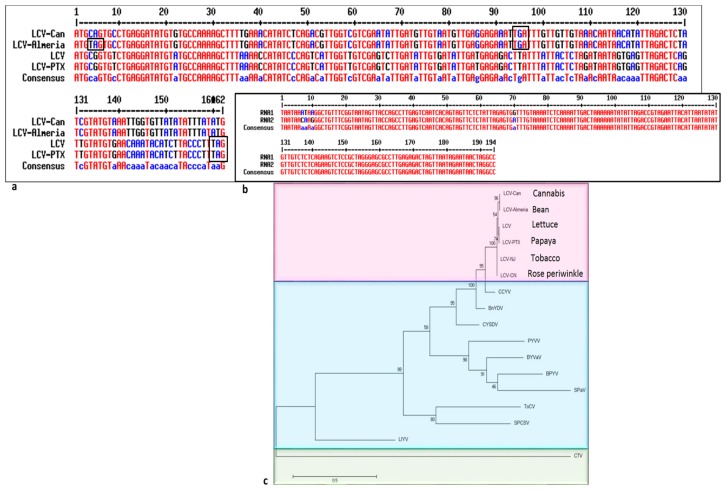
Nucleotide sequence alignment of lettuce chlorosis virus (LCV) P6-like protein and 3′ UTRs of RNA1 and RNA2 fragments and phylogenetic analysis of LCV-Can. (**a**) P6-like putative protein encoded by RNA2 of 4 LCV isolates: LCV-Can, accession No. MK747246, LCV-Almeria, accession No. MG489895, LCV (California), accession No. FJ380119 and LCV-PTX, accession No. KY271956. (**b**) 3′ UTRs of RNA1 and RNA2 fragments of LCV-Can, accession Nos. MK747245 and MK747246. (**c**) Phylogenetic tree analysis based on the coat protein of the following virus isolates: LCV-Can, (MK747246); LCV-Almeria (AUT30585); LCV (California), (YP_003002362); LCV-PTX (ASS35991); LCV-CN (ATQ62183); LCV-NJ (AST35793); cucurbit chlorotic yellows virus (CCYV; AHC06148); bean yellow disorder virus (BnYDV; YP_001816779); cucurbit yellow stunting disorder virus (CYSDV; AGV08356); potato yellow vein virus (PYVV; AMR69001); blackberry yellow vein associated virus (BYVaV; YP_227364); beet pseudo-yellows virus (BPYV; SPN63210); strawberry pallidosis associated virus (SPaV; AAS79680); tomato chlorosis virus (ToCV; AAR15080); sweet potato chlorosis stunt virus (SPCSV; CAD21949); lettuce infectious yellows virus (LIYV; AAA61802) and citrus tristeza virus (CTV; ABB59458).

Regarding the putative encoded proteins, similarity was observed when comparing the putative proteins of LCV-Can with those encoded by other LCV isolates (Table 2). The lowest amino acid sequence similarity was observed between LCV-Can P6-like protein and P6 of the California LCV isolate (Table 2). Alignment of the nucleotide sequence of the putative protein encoded by RNA2 ORF2 of four LCV isolates, showed differences in the stop codon location (Figure 3). Almeria LCV showed a stop codon (TAG) at nucleotide position four, apparently preventing the putative P6 protein translation (accession No. MG489895). LCV-Can sequence showed a stop codon (TGA) at nucleotide position ninety-four, resulting in a putative encoded protein of thirty-one amino acids. California LCV (accession No. FJ380119) and LCV-PTX (accession No. ASS35985) encoded a putative fifty-three amino acid protein (Figure 3a).

### 3.6. Consensus Motifs in LCV-Can

Sequencing of the complete genome revealed that the five conserved nucleotides GAAAT found at the start of the 5′ untranslated regions (UTRs) of RNA1 and RNA2 of the California LCV and of other criniviruses [29,30], were also present at the start of the 5′ UTR of LCV-Can RNA1 and RNA2. However, the 5′ UTRs of LCV-Can showed an additional T at the beginning of the five conserved nucleotides (not attributed to the RACE technique). The rest of the 5′ UTR sequence showed very low percent identity between the two RNAs. Unlike the 5′ UTRs, the 3′ UTR nucleotide sequence of LCV-Can RNA1 and RNA2 were 98% identical (Figure 3b).

Similarities were observed between the LCV proteins P8 and P5.6 [29] of LCV-Can RNA1 and RNA2, respectively and those of the other LCV isolates (Table 2). Interestingly, LCV-Can sequence analysis showed the lack of P4.8 putative protein of LCV [29]. The similarity between the predicted protein of the California LCV ORF1a and that of LCV-Can encompassed the cysteine and histidine catalytic residues, which are located at the same positions: amino acids 406 and 455, respectively [29]. The consensus motifs in the predicted methyl-transferase domain: I, Ia1, Ia2, II, IIa1, III and IV [31,32] were found at amino acids: 556–577, 590–596, 605–612, 613–621, 642–649, 659–687 and 716–736, respectively in both LCV-Can and in California LCV (accession No. FJ380118). Similarly, the six consensus motifs in the predicted helicase domain [32] were found in LCV-Can and in California LCV (accession No. FJ380118) at amino acid positions: 1688–1702, 1761–1771, 1788–1799, 1824–1832, 1910–1927 and 1941–1949. The predicted RdRp protein encoded by ORF1b showed the eight conserved motifs for RNA polymerase of positive strand RNA viruses [32,33] at positions: 186–197, 207–231, 239–254, 269–280, 324–350, 358–367, 394–401 and 412–422, which were also similar to those of California LCV ORF1b (accession No. FJ380118). In addition, the conserved residues of the proteins that comprise the virion capsid were found at similar amino acid positions in LCV-Can and in California LCV (accession No. FJ380119). The putative Hsp70 homolog showed the five ATPase characteristic motifs [34] at amino acids 6–26, 166–180, 192–209, 314–338 and 348–362. The P60 putative protein showed arginine and aspartate at positons 434 and 471, respectively [29]. The putative CP showed the conserved residues serine arginine glycine and aspartate that participate in virion assembly [35], at amino acid positions: 119, 166, 192 and 203, respectively. The putative CPm showed the same conserved amino acids at positions: 348, 392, 422 and 433, respectively.

### 3.7. Phylogenetic Tree Analysis

The CP putative amino acid sequence of six LCV isolates (Table 2) and selected criniviruses served for the phylogenetic analysis. The analysis showed that LCV-Can and the other five LCV isolates are clustered in a clade with cucurbit chlorotic yellows virus (CCYV), bean yellow disorder virus (BnYDV) and cucurbit yellow stunting disorder virus (CYSDV) (Figure 3c).

### 3.8. Whitefly Transmission of LCV-Can in Cannabis Plants and Partial Host Range

**Figure 4:**
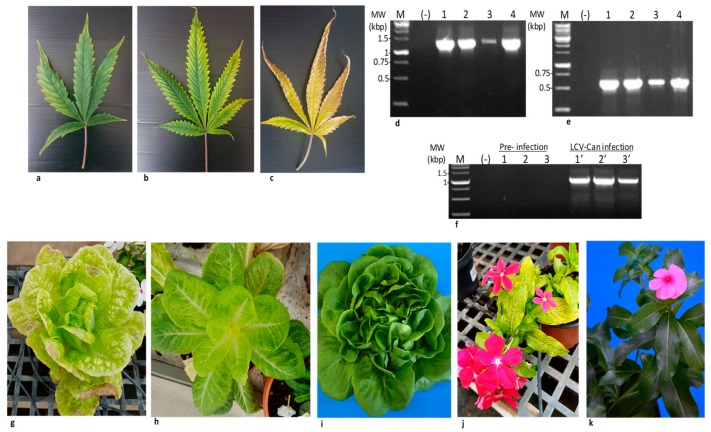
Cannabis lettuce chlorosis virus (LCV-Can) isolate transmitted by *B. tabaci* MEAM1 biotype to un-infected cannabis, lettuce and rose periwinkle plants. (**a**–**c**) LCV-Can infected cannabis leaves at three time points post-viral transmission: 21, 45 and 60 days post-inoculation (dpi), respectively. (**d**,**e**) RT-PCR detection of LCV-Can infected cannabis plants at 35 dpi (**d**) Detection of RNA1 fragment using the primers RNA1-F-7170 and RNA1-R-8433 (Table 3). (**e**) Detection of RNA2 fragment using the primers RNA2-F-7628 and RNA2-R-8189 (Table 3). (**f**) RT-PCR of un-infected and LCV-Can infected Butter-head lettuce (lanes 1 and 1′), Romaine lettuce (lanes 2 and 2′) and rose periwinkle (lanes 3 and 3′) plants, using the primers RNA2-F-6090 and RNA2-R-7094. MW-Molecular weight, M-Marker, (-) Negative control (**g**,**h**) symptomatic LCV-Can infected Butter-head lettuce and Romaine lettuce at 35 dpi. (**i**) Untreated ‘healthy’ Butter head lettuce. (**j**) Symptomatic LCV-Can infected rose periwinkle at 35 dpi. (**k**) Untreated ‘healthy’ rose periwinkle.

LCV-Can infected symptomatic cannabis plants, as confirmed by RT-PCR, served as a source of viral inoculum. Transmission of LCV-Can occurred by naive *B. tabaci* whiteflies (LCV-Can free, propagated on cotton plants) to two known LCV hosts and cannabis plants. The lettuce varieties Butter-head (six out of seven tested plants) and Romaine (six out of seven tested plants), the flowering plant rose periwinkle (five out of seven tested plants) and cannabis plants (seven out of twelve tested plants) showed severe symptoms after four weeks and were positive for the virus as analyzed by RT-PCR (Figure 4, Table 3). In two additional LCV-Can whitefly transmission experiments using twelve cannabis plants in each experiment, which were carried out for 45 and 90 days post-whitefly transmission, all the plants showed severe symptoms and LCV-Can was detected by RT-PCR. Monitoring symptom development of the infected cannabis plants showed that at twenty-one days post-whitefly transmission, disease symptoms were apparently characterized by a pale green interveinal chlorosis of mature leaves, mostly located at the lower-middle part of the plants (Figure 4a). At forty-five days, the pale green areas became clear yellowing streaks (Figure 4b) combined with a drooping foliage appearance and leaf brittleness. The leaves turned completely yellow at sixty days, occasionally accompanied by curled leaf margins and conspicuous necrosis (Figure 4c).

### 3.9. LCV-Can Disease Spread via Shoots

**Figure 5:**
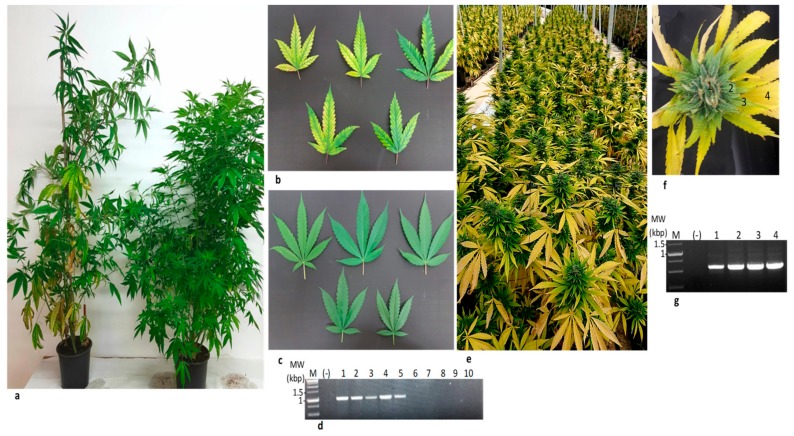
Cannabis lettuce chlorosis virus (LCV) isolate transmitted via shoots.(**a**) Stunted growth of cannabis plant propagated from LCV infected shoots (left) compared to the growth of cannabis plant propagated from un-infected shoots (right). (**b**) Chlorotic leaves of the LCV infected cannabis plant shown in (a). (**c**) Un-infected cannabis leaves. (**d**) RT-PCR detection of LCV-Can infected (lanes 1–5) and un-infected (lanes 6–10) cannabis leaves using the primers RNA2-F-6090 and RNA2-F-7094 (Table 3). (**e**) Symptoms of propagated LCV-can infected plants in an authorized farm (AF). This severe stage was observed one-month post-fertilizer arrest, traditionally done before flower pickup. (**f**) A symptomatic flower of LCV infected propagated cannabis plant dissected to four samples (1–4). (**g**) RT-PCR of LCV infected cannabis-dissected flower (lanes 1–4) using the primers RNA2-F-7628 and RNA2-F-8189 for RNA2 (Table 3). MW-Molecular weight, M-Marker, (-) Negative control.

Cannabis plants that originated from shoots of LCV-Can infected ‘mother plants’ showed disease symptoms, which were more severe than those observed in the original ‘mother plants’ (Figure 5). The symptomatic plants showed conspicuous stunted plant growth with sparse foliage and interveinal chlorosis up to complete leaf yellowing. The differences between the cannabis plants originated from infected and uninfected ‘mother plants’, were distinct and in cannabis AF the damage was drastic. RT-PCR analysis showed the presence of the virus only in the symptomatic plants (Figure 5d, Table 3). Importantly, similar to closteroviruses, LCV-Can disease did not spread via cannabis seeds (Figure S1) [36].

## 4. Discussion

Recent incidences of chlorotic foliage in cannabis plants, attributed to nutrition deprivation, have initiated our study on potential cannabis viral diseases. We have shown by NGS analyses, complemented and validated by RT-PCR amplification and followed by whitefly transmission experiments that the cannabis yellowing disease was indeed caused by the crinivirus LCV. The cannabis isolate of LCV, provisionally named LCV-Can, demonstrated high nucleotide sequence identity and amino acid sequence similarity to the reported LCV isolates from lettuce, bean, papaya, tobacco and rose periwinkle that were deposited in GenBank database. The 3′ UTRs of LCV-Can RNA1 and RNA2 were 98% identical, indicating that similar to other criniviruses, excluding lettuce infectious yellows virus (LIYV), LCV-Can replication of RNA1 and RNA2 is synchronous [37]. Importantly, typically to *crinivirus* species, LCV-Can was transmitted successfully from infected cannabis plants to un-infected ‘healthy’ plants by the whitefly vector *B. tabaci* MEAM1 biotype. Similar LCV-Can disease symptoms developed in the inoculated plants 30–40 days post the whitefly transmission. Criniviruses are known to be transmitted by the whitefly vector in a semi-persistent manner [38]. Cannabis strains are diverse, each having a characteristic cannabinoid content [39]. In cannabis AF, plants are bred for the establishment of unique and specific genetic verities that are characterized by their cannabinoid composition. Following the breeding stage, selected genetic source plants are kept separately and propagated continuously as ‘mother plants’ for shoot generation in the nursery to produce the next growing cycle. Therefore, the most important mode of infection that could occur widely in cannabis AF is via shoots from LCV-Can infected ‘mother plants’ that serve as an inoculum source for disease spread. Our results, implicating LCV-Can in the chlorotic symptoms observed in cannabis plants, are important for cannabis AF that should now analyze ‘mother plants’ for LCV-Can infection before plant propagation.

In recent years, whitefly transmitted viruses, including criniviruses, have become a global problem in a wide range of crops [16,40]. The worldwide spread of the viruses occurred concomitant to host range expansion [17,41,42]. The parallel spread of *B. tabaci* MEAM1 biotype [43,44,45], could be the root cause of the LCV-Can disease occurred in cannabis plants in Israel. Phylogenetic analysis based on LCV-CP revealed that the examined LCV isolates comprised part of Group-2 of the criniviruses, as classified by results of phylogenetic analysis based on 1a/1b polyprotein [17]. Although clustered in a clade with CCYV and CYSDV, the LCV isolates do not infect cucurbits but the viruses share the *B. tabaci* vector. Interestingly, we have observed recently increased occurrence of CCYV and CYSDV in cucurbits in Israel [18] indicating a possible rise in *B. tabaci* spread.

Disease symptoms characteristic to criniviruses include in general yellow spots that occur in older leaves and could develop to interveinal chlorosis, leaf thickness and brittleness [46]. Similar symptoms were observed in LCV infected lettuce plants [38]. However, in LCV-Can infected cannabis plants, which were grown in greenhouses in Israel, occasional necrosis was observed as well. Recently it has been shown that P23 putative protein encoded by RNA1 of California LCV, induced temperature-dependent necrosis in *Nicotiana benthamiana* agro-infiltrated plants via suppression of RNA silencing [47]. The temperatures that were permissive for necrosis development began at 21°C. It is possible therefore, that LCV-Can putative P23 conferred the necrotic phenotype observed in the symptomatic cannabis plants.

Various plants belonging to the rosid clade show susceptibility to LCV infection [38]. *C. sativa* belongs to fabidae subclade of the rosid clade [48]. Interestingly, LCV-Can RNAs shared 99.6% nucleotide sequence identity with RNAs of Almeria LCV, isolated from the bean *Phaseolus vulgaris*, which also belongs to the fabidae subclade of the rosid clade. Accordingly, LCV-Can RNA1 and RNA2 shared lower nucleotide sequence identity with RNAs of California LCV, LCV-PTX, LCV CN and LCV-NJ that were isolated from plants belonging to the asterid clade (*Lactuca sativa*, *Catharanthus roseus*, *Nicotiana tabacum*) or in the case of LCV-PTX the host plant (*Carica papaya*) belongs to the malvidae subclade of the rosid clade. Host preference for specific crinivirus isolates is often attributed to the virus capabilities of replication, movement in the plants and/or suppression of plant immune responses, e.g., RNA silencing [49,50]. LCV-Can movement in cannabis plants could be affected by the viral P6-like protein, which is putatively a 3.4 kDa protein (thirty-one amino acids) encoded by LCV-Can RNA2. P6 was shown to be associated with cell-to-cell movement capabilities of the closterovirus Beet yellows virus [51]. Further studies are necessary to confirm the occurrence of any specific adaptation of LCV-Can to cannabis plants. Whether there is a possible effect of host plants on the infecting crinivirus sequence is an intriguing question. This might occur due to recombination events between criniviruses co-infecting a host [30,52].

## 5. Conclusions

Symptoms of chlorotic foliage and complete leaf yellowing observed in cannabis plants, commonly attributed to nutrition deprivation, were the outcome of infection by the crinivirus LCV-Can. Testing ‘mother plants’, used for cannabis plant propagation via shoots, for the presence of LCV-Can would be an important step towards mitigation of the viral cannabis yellowing disease. Importantly, future strategies for alleviating the viral disease spread should include screening for cannabis strains resistant to LCV-Can.

## Figures and Tables

**Table 1:** Next generation sequencing results of constructed libraries.

		Small RNA				Total RNA			
Reference Genome ^a^	Complete Genome (nt)	No. Contigs ^b^	Coverage ^c^ (%)	Depth ^d^	Identity ^e^	No. Contigs	Coverage (%)	Depth	Identity
*Lettuce chlorosis virus* (LCV) **RNA1**	8591	22	1054 (12.3%)	5.7	97.25	39	7103 (82.7%)	2671.7	89.78
*Lettuce chlorosis virus* (LCV) **RNA2**	8556	38	2091 (24.4%)	5.3	97.32	52	6722 (78.6%)	2499.3	92.61

^a^ GenBank reference genome of LCV (accession Nos. of RNA1, FJ380118 and RNA2, FJ380119), with which the assembled contigs had high similarity. ^b^ No. of contigs aligned to the reference genome. ^c^ Percentage of LCV reference genome covered by the assembled contigs. ^d^ The average of the No. of times each nucleotide of the reference genome was covered by the identified sequences. ^e^ The average (%) of nucleotide sequence identity of all contigs with the GenBank reference genome sequence.

**Table 2:** Comparison (% identity) between nucleotide sequence of whole genome and the deduced amino acids of putative encoded proteins of LCV-Can and other LCV isolates.

Viruses ^a^	LCV-Can	LCV-Almeria	LCV (California)	LCV-PTX	LCV-CN	LCV-NJ
RNA1 Putative proteins (aa.)	Whole genome (nt.)	99	89	89	85	85
	1a	99	94	94	91	91
	RdRp	100	99	99	99	99
	P8	-	74	74	55	57
	P23	-	75	75	77	77
RNA2 Putative proteins (aa.)	Whole genome (nt.)	99	90	90	89	89
	P5.6	100	92	92	91	91
	P6-like	-	52	52	-	-
	HSP70	100	98	98	99	98
	P6.4	100	98	98	98	98
	P60	99	97	97	95	95
	P9	100	96	96	96	96
	CP	100	96	95	98	98
	CPm	99	96	96	92	92
	P27	99	95	97	97	97

^a^ LCV-Can (MK747245, MK747246); LCV-Almeria (MG489894, MG489895); LCV (California) (FJ380118, FJ380119); LCV-PTX (KY271955, KY271956); LCV-CN (KY430285, KY430286); LCV-NJ (KX685958, KX685959).

**Table 3:** Primer pairs used for diagnosis of lettuce chlorosis virus (LCV) in cannabis plants.

* Set No.	** Orientation	Name-Position	Sequence (5′-3′)
8	F	RNA1-F-7170	TCACAGCCGAGATCAACAGA
8	C	RNA1-R-8433	GTTACCAGCCTTGAGTCAATCA
12	F	RNA2-F-6090	TCATCTTCAGGCCAAACACGG
12	C	RNA2-R-7094	TCCACCTAATCCGATTCCAC
13	F	RNA2-F-7628	GCAGGTCATGACGTCAGATTT
13	C	RNA2-R-8189	TGAACAATCACTACAGGTTTGG

* Primer set numbers are based on Table S1. ** F = forward, C = complement.

## Supplementary Materials

The following are available online at https://www.mdpi.com/1999-4915/11/9/802/s1. Table S1: Primer pairs used for sequencing cannabis lettuce chlorosis virus (LCV) isolate, Table S2: Overlapping primer pairs used for sequencing cannabis lettuce chlorosis virus (LCV) isolate, Figure S1: Analysis of cannabis lettuce chlorosis virus (LCV-Can) seed transmission.

## References

[1] Rubin V. (1975). The ”Ganja Vision” in Jamaica. Cannabis and Culture.

[2] Berman P., Futoran K., Lewitus G.M., Mukha D., Benami M., Shlomi T., Meiri D. (2018). A new ESI-LC/MS approach for comprehensive metabolic profiling of phytocannabinoids in Cannabis. Sci. Rep..

[3] Russo E.B., Hohmann A.G. (2013). Role of cannabinoids in pain management. Comprehensive Treatment of Chronic Pain by Medical, Interventional, and Integrative Approaches.

[4] Grund J.-P.C., Breeksema J.J. (2017). Drug policy in the Netherlands. European Drug Policies: The Ways of Reform.

[5] Zaami S., Di Luca A., Di Luca N., Vergallo G.M. (2018). Medical use of cannabis: Italian and European legislation. Eur. Rev. Med. Pharmacol. Sci..

[6] Room R. (2014). Legalizing a market for cannabis for pleasure: Colorado, Washington, Uruguay and beyond. Addiction.

[7] Hughes B., Wiessing L., Jarlais D.D., Griffiths P. (2018). Could cannabis liberalisation lead to wider changes in drug policies and outcomes?. Int. J. Drug Policy.

[8] King K.C., Lively C.M. (2012). Does genetic diversity limit disease spread in natural host populations?. Heredity.

[9] Alonso M., Borja M. (2005). High incidence of *Pelargonium line pattern virus* infecting asymptomatic *Pelargonium* spp. in Spain. Eur. J. Plant Pathol..

[10] Röder K. (1941). Einige Untersuchungen über ein an Hanf (*Cannabis sativa* L.) auftretendes Virus. Faserforschung.

[11] Ceapoiu N. (1958). Cinepa: Estudiu Monografic. Institutul de Cercetari Agronomice.

[12] McPartland J.M., Clarke R.C., Watson D.P. (2000). Hemp Diseases and Pests: Management and Biological Control—An Advanced Treatise.

[13] Bektas A., Hardwick K.M., Waterman K., Kristof J. (2019). The Occurrence of Hop Latent Viroid in *Cannabis sativa* with symptoms of Cannabis Stunting Disease in California. Plant Dis..

[14] Warren J., Mercado J., Grace D. (2019). The occurrence of Hop latent viroid causing disease in *Cannabis sativa* in California. Plant Dis..

[15] Ziegler A., Matoušek J., Steger G., Schubert J. (2012). Complete sequence of a cryptic virus from hemp (*Cannabis sativa*). Arch. Virol..

[16] Navas-Castillo J., Fiallo-Olivé E., Sánchez-Campos S. (2011). Emerging Virus Diseases Transmitted by Whiteflies. Annu. Rev. Phytopathol..

[17] Tzanetakis I.E., Martin R.R., Wintermantel W.M. (2013). Epidemiology of criniviruses: An emerging problem in world agriculture. Front. Microbiol..

[18] Luria N., Smith E., Sela N., Koren A., Lachman O., Dombrovsky A. (2019). Insights Into a Watermelon Virome Contribute to Monitoring Distribution of Whitefly-Borne Viruses. Phytobiomes J..

[19] Sela N., Luria N., Dombrovsky A. (2012). Genome Assembly of Bell Pepper Endornavirus from Small RNA. J. Virol..

[20] Stocks M.B., Moxon S., Mapleson D., Woolfenden H.C., Mohorianu I., Folkes L., Schwach F., Dalmay T., Moulton V. (2012). The UEA sRNA workbench: A suite of tools for analysing and visualizing next generation sequencing microRNA and small RNA datasets. Bioinformatics.

[21] Li H., Handsaker B., Wysoker A., Fennell T., Ruan J., Homer N., Marth G., Abecasis G., Durbin R. (2009). The Sequence Alignment/Map format and SAMtools. Bioinformatics.

[22] Zheng Y., Gao S., Padmanabhan C., Li R., Galvez M., Gutierrez D., Fuentes S., Ling K.-S., Kreuze J., Fei Z. (2017). VirusDetect: An automated pipeline for efficient virus discovery using deep sequencing of small RNAs. Virology.

[23] Langmead B., Salzberg S.L. (2012). Fast gapped-read alignment with Bowtie 2. Nat. Methods.

[24] Zerbino D.R., Birney E. (2008). Velvet: Algorithms for de novo short read assembly using de Bruijn graphs. Genome Res..

[25] Li H., Durbin R. (2009). Fast and accurate short read alignment with Burrows–Wheeler transform. Bioinformatics.

[26] Frohman M.A. (1990). RACE: Rapid amplification of cDNA ends. PCR Protocols: A Guide to Methods and Applications.

[27] Edgar R.C. (2004). MUSCLE: Multiple sequence alignment with high accuracy and high throughput. Nucleic Acids Res..

[28] Tamura K., Stecher G., Peterson D., Filipski A., Kumar S. (2013). MEGA6: Molecular Evolutionary Genetics Analysis Version 6.0. Mol. Boil. Evol..

[29] Salem N.M., Chen A.Y., Tzanetakis I.E., Mongkolsiriwattana C., Ng J.C. (2009). Further complexity of the genus *Crinivirus* revealed by the complete genome sequence of *Lettuce chlorosis* virus (LCV) and the similar temporal accumulation of LCV genomic RNAs 1 and 2. Virology.

[30] Ruiz L., Simón A., García C., Velasco L., Janssen D. (2018). First natural crossover recombination between two distinct species of the family *Closteroviridae* leads to the emergence of a new disease. PLoS ONE.

[31] Rozanov M.N., Koonin E.V., Gorbalenya A.E. (1992). Conservation of the putative methyltransferase domain: A hallmark of the ‘Sindbis-like’ supergroup of positive-strand RNA viruses. J. Gen. Virol..

[32] Tzanetakis I., Reed J., Martin R. (2005). Nucleotide sequence, genome organization and phylogenetic analysis of Strawberry pallidosis associated virus, a new member of the genus *Crinivirus*. Arch. Virol..

[33] Koonin E.V. (1991). The phylogeny of RNA-dependent RNA polymerases of positive-strand RNA viruses. J. Gen. Virol..

[34] Bork P., Sander C., Valencia A. (1992). An ATPase domain common to prokaryotic cell cycle proteins, sugar kinases, actin, and hsp70 heat shock proteins. Proc. Natl. Acad. Sci. USA.

[35] Alzhanova D.V., Napuli A.J., Creamer R., Dolja V.V. (2001). Cell-to-cell movement and assembly of a plant closterovirus: Roles for the capsid proteins and Hsp70 homolog. EMBO J..

[36] King A.M.Q., Adams M.J., Carstens E.B., Lefkowitz E.J. (2012). Ninth Report of the International Committee on Taxonomy of Viruses.

[37] Klaassen V.A., Boeshore M.L., Koonin E.V., Tian T., Falk B.W. (1995). Genome Structure and Phylogenetic Analysis of Lettuce Infectious Yellows Virus, a Whitefly-Transmitted, Bipartite Closterovirus. Virology.

[38] Duffus J.E., Liu H.-Y., Wisler G.C., Li R. (1996). Lettuce chlorosis virus—A new whitefly-transmitted closterovirus. Eur. J. Plant Pathol..

[39] Hillig K.W., Mahlberg P.G. (2004). A chemotaxonomic analysis of cannabinoid variation in *Cannabis* (Cannabaceae). Am. J. Bot..

[40] Gilbertson R.L., Batuman O., Webster C.G., Adkins S. (2015). Role of the Insect Supervectors *Bemisia tabaci* and *Frankliniella occidentalis* in the Emergence and Global Spread of Plant Viruses. Annu. Rev. Virol..

[41] Ruiz M.L., Simon A., Garcia M.C., Janssen D. (2014). First Report of Lettuce chlorosis virus Infecting Bean in Spain. Plant Dis..

[42] Alabi O.J., Al Rwahnih M., Jifon J.L., Sétamou M., Brown J.K., Gregg L., Park J.-W. (2017). A mixed infection of Lettuce chlorosis virus, Papaya ringspot virus, and Tomato yellow leaf curl virus-IL detected in a Texas papaya orchard affected by a virus-like disease outbreak. Plant Dis..

[43] De Barro P.J., Liu S.-S., Boykin L.M., Dinsdale A.B. (2011). Bemisia tabaci: A Statement of Species Status. Annu. Rev. Èntomol..

[44] Hadjistylli M., Roderick G.K., Brown J.K. (2016). Global Population Structure of a Worldwide Pest and Virus Vector: Genetic Diversity and Population History of the *Bemisia tabaci* Sibling Species Group. PLoS ONE.

[45] Ghanim M. (2014). A review of the mechanisms and components that determine the transmission efficiency of *Tomato yellow leaf curl virus* (Geminiviridae; *Begomovirus*) by its whitefly vector. Virus Res..

[46] Loebenstein G., Lecoq H. (2012). Viruses and Virus Diseases of Vegetables in the Mediterranean Basin.

[47] Kubota K., Ng J. (2016). Lettuce chlorosis virus P23 Suppresses RNA Silencing and Induces Local Necrosis with Increased Severity at Raised Temperatures. Phytopathology.

[48] Cantino P.D., Doyle J.A., Graham S.W., Judd W.S., Olmstead R.G., Soltis D.E., Soltis P.S., Donoghue M.J., Wang W., Chen Z.-D. (2007). Towards a phylogenetic nomenclature of Tracheophyta. TAXON.

[49] Kiss Z.A., Medina V., Falk B.W. (2013). Crinivirus replication and host interactions. Front. Microbiol..

[50] Diaz-Pendon J.A., Ding S.-W. (2008). Direct and Indirect Roles of Viral Suppressors of RNA Silencing in Pathogenesis. Annu. Rev. Phytopathol..

[51] Alzhanova D.V., Hagiwara Y., Peremyslov V.V., Dolja V.V. (2000). Genetic Analysis of the Cell-to-Cell Movement of Beet Yellows Closterovirus. Virology.

[52] Zhao X., Zhu M., Wu Q., Zhang J., Xu Y., Tao X. (2018). Complete genome sequence of a lettuce chlorosis virus isolate from China and genome recombination/rearrangement analysis. Arch. Virol..

