# Supplementary material for: Lettuce Chlorosis Virus Disease: A New Threat to Cannabis Production
Source: Viruses. 2019 Aug 29;11(9):802. doi: 10.3390/v11090802 (PMC6784094; doi:10.3390/v11090802)
Supplement: Supplementary file 1 [file viruses-11-00802-s001.zip › Supplementary file.docx]

**Table S1.** Primer pairs used for sequencing cannabis lettuce chlorosis virus (LCV) isolate.

| **Set No.** | ***Orientation** | **Name-position** | **Sequence (5’-3’)** |
| --- | --- | --- | --- |
| 1 | F | RNA1-F-68 | CCTTAATGGCCGTGATGTCG |
| 1 | R | RNA1-R-1572 | ATCAGGCACACAGACTCGAA |
| 2 | F | RNA1-F-4587 | ACAATTCTCTATGCTCAAACCGT |
| 2 | R | RNA1-R-5941 | GTGATCACTGACAAGATGTTCGA |
| 3 | F | RNA2-F-2238 | GTCTCGGTCCCAGCGGATTA |
| 3 | R | RNA2-R-2954 | TGTCAGTTTCTTTTGTAACCCT |
| 4 | F | RNA2-F-5107 | TCGTACACTCCGTCTGATGA |
| 4 | R | RNA2-R-5925 | CACTCTGCAGATCAAAGGGC |
| 5 | F | RNA1-F-1900 | TGTTCACCAGTTGTAGACCT |
| 5 | R | RNA1-R-2695 | TATGGATTGCGCCTGAGAGA |
| 6 | F | RNA1-F-2756 | ACACTCCATCTCTTTTCGGT |
| 6 | R | RNA1-R-4258 | AGGTCTGTATGTGTGAAAGCA |
| 7 | F | RNA1-F-5473 | CGGGTTGCTCACACAGTTTG |
| 7 | R | RNA1-R-6687 | TTGTGAATCTCTTCTACTCCC |
| 8 | F | RNA1-F-7170 | TCACAGCCGAGATCAACAGA |
| 8 | R | RNA1-R-8433 | GTTACCAGCCTTGAGTCAATCA |
| 9 | F | RNA2-F-949 | ACTTTAAACTGTGTCGCCGT |
| 9 | R | RNA2-R-2011 | ACGATCTTAAAAGATGGGTTGG |
| 10 | F | RNA2-F-2892 | CATGCTCAAGAAGACACGGG |
| 10 | R | RNA2-R-4250 | TTCAGAGAATGTGTGGAAGC |
| 11 | F | RNA2-F-4368 | AGAGTCCAGAGATCAAAGTAGT |
| 11 | R | RNA2-R-5828 | CTGCTGATGAGTTCTTGGCA |
| 12 | F | RNA2-F-6090 | TCATCTTCAGGCCAAACACGG |
| 12 | R | RNA2-R-7094 | TCCACCTAATCCGATTCCAC |
| 13 | F | RNA2-F-7628 | GCAGGTCATGACGTCAGATTT |
| 13 | R | RNA2-R-8189 | TGAACAATCACTACAGGTTTGG |
| 14 | F | RNA1-F-1263 | GTTTGGGTCCTGTGGCAATT |
| 14 | R | RNA1-R-2695 | TATGGATTGCGCCTGAGAGA |
| 15 | F | RNA1-F-2376 | AGACATGATGAACGGGAGCT |
| 15 | R | RNA1-R-4258 | AGGTCTGTATGTGTGAAAGCA |
| 16 | F | RNA1-F-3987 | TGGAATTGTGACAGCTCCCA |
| 16 | R | RNA1-R-5941 | GTGATCACTGACAAGATGTTCGA |
| 17 | F | RNA1-F-6473 | CCAGTTGTGCCCGATTTGAA |
| 17 | R | RNA1-R-8433 | GTTACCAGCCTTGAGTCAATCA |
| 18 | F | RNA2-F-1770 | GAACCCCTCTAATCCCCTCC |
| 18 | R | RNA2-R-2954 | TGTCAGTTTCTTTTGTAACCCT |
| 19 | F | RNA2-F-2593 | ATTCAAGTGGCAGGGAGTCA |
| 19 | R | RNA2-R-4250 | TTCAGAGAATGTGTGGAAGC |
| 20 | F | RNA2-F-3937 | TCAACCGGGATCTCTGTTCA |
| 20 | R | RNA2-R-5828 | CTGCTGATGAGTTCTTGGCA |
| 21 | F | RNA2-F-5613 | CGTAACAAGAGAAAGCGAGGG |
| 21 | R | RNA2-R-7094 | TCCACCTAATCCGATTCCAC |
| 22 | F | RNA2-F-6784 | GCGTGTTCCAAAGCATCTCA |
| 22 | R | RNA2-R-8189 | TGAACAATCACTACAGGTTTGG |
| RACE 5’ | R | RNA1-R-312 | GGTAGCCTTTAGAAGAAGGC |
| RACE 5’ | R | RNA2-R-250 | CTGAACTGGTGTCAACGATCATGCG |
| RACE 3’ | F | RNA1-F-7882 | CAATTTAAAAACCGGTCAAG |
| RACE 3’ | F | RNA2-F-8220 | TCAAGCAGACTTCTCAATCA |

*F=forward, C=complement

**Table S2.** Overlapping primer pairs used for sequencing cannabis lettuce chlorosis virus (LCV) isolate.

| **Primer set No.** | ***Orientation** | **Name-position** | **Sequence (5'-3')** |
| --- | --- | --- | --- |
| 1 | F | RNA1-F-1 | TGAAATCAAACTTTCCTTCGTA |
| 1 | C | RNA1-R-1572 | ATCAGGCACACAGACTCGAA |
| 2 | F | RNA1-F-1263 | GTTTGGGTCCTGTGGCAATT |
| 2 | C | RNA1-R-2695 | TATGGATTGCGCCTGAGAGA |
| 3 | F | RNA1-F-2376 | AGACATGATGAACGGGAGCT |
| 3 | C | RNA1-R-4258 | AGGTCTGTATGTGTGAAAGCA |
| 4 | F | RNA1-F-3987 | TGGAATTGTGACAGCTCCCA |
| 4 | C | RNA1-R-5941 | GTGATCACTGACAAGATGTTCGA |
| 5 | F | RNA1-F-5473 | CGGGTTGCTCACACAGTTTG |
| 5 | C | RNA1-R-6687 | TTGTGAATCTCTTCTACTCCC |
| 6 | F | RNA1-F-6473 | CCAGTTGTGCCCGATTTGAA |
| 6 | C | RNA1-R-3'End | GTTAATAGAATAACTAGGCC |
| 7 | F | RNA2-F-1 | TGAAATTTTCCACGGTTTCCC |
| 7 | C | RNA2-R-2011 | ACGATCTTAAAAGATGGGTTGG |
| 8 | F | RNA2-F-1770 | GAACCCCTCTAATCCCCTCC |
| 8 | C | RNA2-R-2954 | TGTCAGTTTCTTTTGTAACCCT |
| 9 | F | RNA2-F-2892 | CATGCTCAAGAAGACACGGG |
| 9 | C | RNA2-R-4250 | TTCAGAGAATGTGTGGAAGC |
| 10 | F | RNA2-F-3937 | TCAACCGGGATCTCTGTTCA |
| 10 | C | RNA2-R-5925 | CACTCTGCAGATCAAAGGGC |
| 11 | F | RNA2-F-5613 | CGTAACAAGAGAAAGCGAGGG |
| 11 | C | RNA2-R-7094 | TCCACCTAATCCGATTCCAC |
| 12 | F | RNA2-F-6784 | GCGTGTTCCAAAGCATCTCA |
| 12 | C | RNA2-R-3'End | GTTAATAGAATAACTAGGCC |

*F=forward, C=complement

**Figure S1**

**Figure** **S1** Analysis of cannabis lettuce chlorosis virus (LCV-Can) seed transmission.

**(a)** LCV-Can in seeds of symptomatic cannabis plants. Lanes 1-5, RT-PCR with primer set No. 8, (Table S1). Lanes 6-10, RT-PCR with primer set No. 13 (Table S1). Lanes 4,9- symptomatic cannabis plants. Lanes 1-3, 6-8-seeds of symptomatic cannabis plants,100 seeds in each lane. Lanes 5,10- no template control (NTC). **(b**) LCV-Can seed to seedling transmission. Lanes 1-4, RT-PCR with primer set No. 8, (Table S1). Lanes 5-8, RT-PCR with primer set No. 13 (Table S1). Lanes 1,5- symptomatic cannabis plants. Lanes 2,6- pooled 10 seeds of the symptomatic plant. Lanes 3,7- pooled 8 seedlings of the sown seeds from the symptomatic plant. Lanes 4,8- no template control (NTC).
