# Supplementary material for: Lettuce Chlorosis Virus Disease: A New Threat to Cannabis Production
Source: Viruses. 2019 Aug 29;11(9):802. doi: 10.3390/v11090802 (PMC6784094; doi:10.3390/v11090802)
Supplement: Supplementary file 1 [file viruses-11-00802-s001.zip › Supplementary file.pdf]

Table S1. Primer pairs used for sequencing cannabis lettuce chlorosis virus (LCV) isolate.

Set No.	*Orientation	Name-position	Sequence (5′-3′)
1	F	RNA1-F-68	CCTTAATGGCCGTGATGTCG
1	R	RNA1-R-1572	ATCAGGCACACAGACTCGAA
2	F	RNA1-F-4587	ACAATTCTCTATGCTCAAACCGT
2	R	RNA1-R-5941	GTGATCACTGACAAGATGTTCGA
3	F	RNA2-F-2238	GTCTCGGTCCCAGCGGATTA
3	R	RNA2-R-2954	TGTCAGTTTCTTTTGTAACCCT
4	F	RNA2-F-5107	TCGTACACTCCGTCTGATGA
4	R	RNA2-R-5925	CACTCTGCAGATCAAAGGGC
5	F	RNA1-F-1900	TGTTCACCAGTTGTAGACCT
5	R	RNA1-R-2695	TATGGATTGCGCCTGAGAGA
6	F	RNA1-F-2756	ACACTCCATCTCTTTTCGGT
6	R	RNA1-R-4258	AGGTCTGTATGTGTGAAAGCA
7	F	RNA1-F-5473	CGGGTTGCTCACACAGTTTG
7	R	RNA1-R-6687	TTGTGAATCTCTTCTACTCCC
8	F	RNA1-F-7170	TCACAGCCGAGATCAACAGA
8	R	RNA1-R-8433	GTTACCAGCCTTGAGTCAATCA
9	F	RNA2-F-949	ACTTTAAACTGTGTCGCCGT
9	R	RNA2-R-2011	ACGATCTTAAAAGATGGGTTGG
10	F	RNA2-F-2892	CATGCTCAAGAAGACACGGG
10	R	RNA2-R-4250	TTCAGAGAATGTGTGGAAGC
11	F	RNA2-F-4368	AGAGTCCAGAGATCAAAGTAGT
11	R	RNA2-R-5828	CTGCTGATGAGTTCTTGGCA
12	F	RNA2-F-6090	TCATCTTCAGGCCAAACACGG
12	R	RNA2-R-7094	TCCACCTAATCCGATTCCAC
13	F	RNA2-F-7628	GCAGGTCATGACGTCAGATTT
13	R	RNA2-R-8189	TGAACAATCACTACAGGTTTGG
14	F	RNA1-F-1263	GTTTGGGTCCTGTGGCAATT
14	R	RNA1-R-2695	TATGGATTGCGCCTGAGAGA
15	F	RNA1-F-2376	AGACATGATGAACGGGAGCT
15	R	RNA1-R-4258	AGGTCTGTATGTGTGAAAGCA
16	F	RNA1-F-3987	TGGAATTGTGACAGCTCCCA
16	R	RNA1-R-5941	GTGATCACTGACAAGATGTTCGA
17	F	RNA1-F-6473	CCAGTTGTGCCCGATTTGAA
17	R	RNA1-R-8433	GTTACCAGCCTTGAGTCAATCA
18	F	RNA2-F-1770	GAACCCCTCTAATCCCCTCC
18	R	RNA2-R-2954	TGTCAGTTTCTTTTGTAACCCT
19	F	RNA2-F-2593	ATTCAAGTGGCAGGGAGTCA
19	R	RNA2-R-4250	TTCAGAGAATGTGTGGAAGC
20	F	RNA2-F-3937	TCAACCGGGATCTCTGTTCA
20	R	RNA2-R-5828	CTGCTGATGAGTTCTTGGCA
21	F	RNA2-F-5613	CGTAACAAGAGAAAGCGAGGG
21	R	RNA2-R-7094	TCCACCTAATCCGATTCCAC
22	F	RNA2-F-6784	GCGTGTTCCAAAGCATCTCA
22	R	RNA2-R-8189	TGAACAATCACTACAGGTTTGG
RACE 5′	R	RNA1-R-312	GGTAGCCTTTAGAAGAAGGC
RACE 5′	R	RNA2-R-250	CTGAACTGGTGTCAACGATCATGCG
RACE 3′	F	RNA1-F-7882	CAATTTAAAAACCGGTCAAG
RACE 3′	F	RNA2-F-8220	TCAAGCAGACTTCTCAATCA

*F=forward, C=complement

Table S2. Overlapping primer pairs used for sequencing cannabis lettuce chlorosis virus (LCV) isolate.

Primer set No.	*Orientation	Name-position	Sequence (5'-3')
1	F	RNA1-F-1	TGAAATCAAACTTTCCTTCGTA
1	C	RNA1-R-1572	ATCAGGCACACAGACTCGAA
2	F	RNA1-F-1263	GTTTGGGTCCTGTGGCAATT
2	C	RNA1-R-2695	TATGGATTGCGCCTGAGAGA
3	F	RNA1-F-2376	AGACATGATGAACGGGAGCT
3	C	RNA1-R-4258	AGGTCTGTATGTGTGAAAGCA
4	F	RNA1-F-3987	TGGAATTGTGACAGCTCCCA
4	C	RNA1-R-5941	GTGATCACTGACAAGATGTTCGA
5	F	RNA1-F-5473	CGGGTTGCTCACACAGTTTG
5	C	RNA1-R-6687	TTGTGAATCTCTTCTACTCCC
6	F	RNA1-F-6473	CCAGTTGTGCCCGATTTGAA
6	C	RNA1-R-3'End	GTTAATAGAATAACTAGGCC
7	F	RNA2-F-1	TGAAATTTTCCACGGTTTCCC
7	C	RNA2-R-2011	ACGATCTTAAAAGATGGGTTGG
8	F	RNA2-F-1770	GAACCCCTCTAATCCCCTCC
8	C	RNA2-R-2954	TGTCAGTTTCTTTTGTAACCCT
9	F	RNA2-F-2892	CATGCTCAAGAAGACACGGG
9	C	RNA2-R-4250	TTCAGAGAATGTGTGGAAGC
10	F	RNA2-F-3937	TCAACCGGGATCTCTGTTCA
10	C	RNA2-R-5925	CACTCTGCAGATCAAAGGGC
11	F	RNA2-F-5613	CGTAACAAGAGAAAGCGAGGG
11	C	RNA2-R-7094	TCCACCTAATCCGATTCCAC
12	F	RNA2-F-6784	GCGTGTTCCAAAGCATCTCA
12	C	RNA2-R-3'End	GTTAATAGAATAACTAGGCC

*F=forward, C=complement

Figure S1

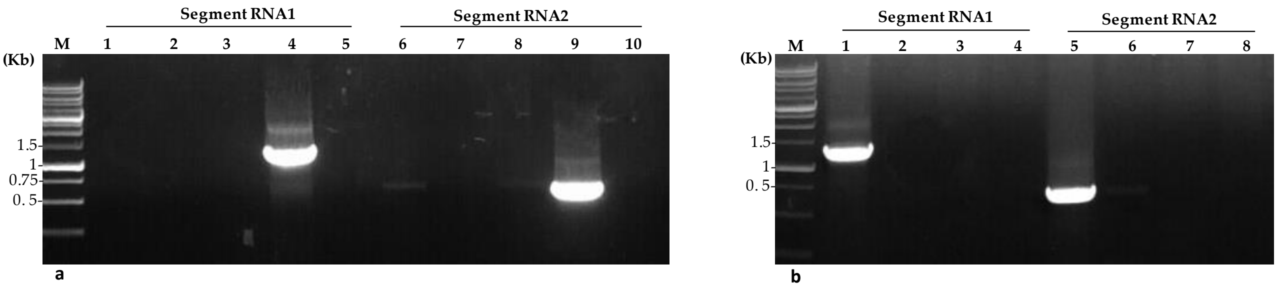

Figure S1 Analysis of cannabis lettuce chlorosis virus (LCV-Can) seed transmission. **(a)** LCV-Can in seeds of symptomatic cannabis plants. Lanes 1-5, RT-PCR with primer set No. 8, (Table S1). Lanes 6-10, RT-PCR with primer set No. 13 (Table S1). Lanes 4,9- symptomatic cannabis plants. Lanes 1-3, 6-8-seeds of symptomatic cannabis plants,100 seeds in each lane. Lanes 5,10- no template control (NTC). **(b)** LCV-Can seed to seedling transmission. Lanes 1-4, RT-PCR with primer set No. 8, (Table S1). Lanes 5-8, RT-PCR with primer set No. 13 (Table S1). Lanes 1,5- symptomatic cannabis plants. Lanes 2,6- pooled 10 seeds of the symptomatic plant. Lanes 3,7- pooled 8 seedlings of the sown seeds from the symptomatic plant. Lanes 4,8- no template control (NTC).
