# Supplementary figures and images for: Lettuce Chlorosis Virus Disease: A New Threat to Cannabis Production
Source: Viruses. 2019 Aug 29;11(9):802. doi: 10.3390/v11090802 (PMC6784094; doi:10.3390/v11090802)

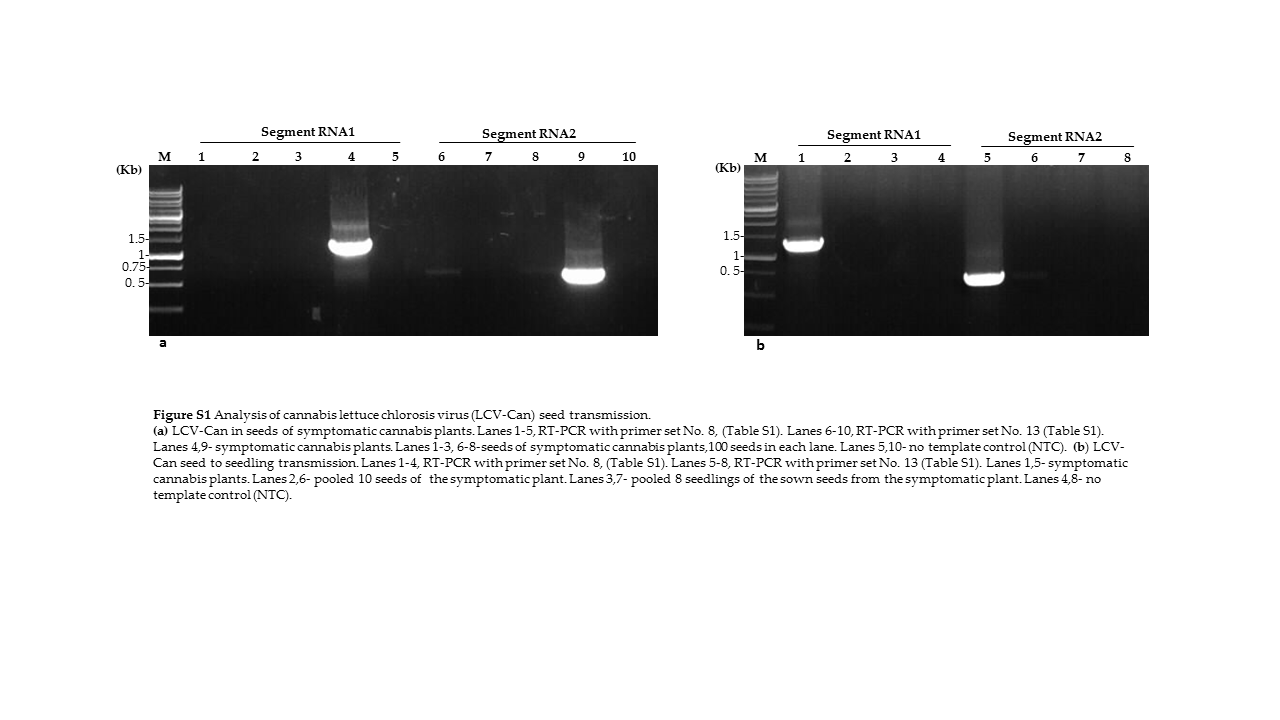

Supplement: Supplementary file 1 [file viruses-11-00802-s001.zip › Figure S1.tif]
